# Sonorheometry to detect traumatic induced coagulopathy during initial management of trauma patients: an observational, multi-center study

**DOI:** 10.1186/s13049-026-01610-8

**Published:** 2026-04-15

**Authors:** Gary Duclos, Pierre Antoine Seube Remy, Mathieu Willig, Mohamed Boucekine, Charles Deregnaucourt, Charlotte Grosdidier, Paul Leblanc, Delphine Garrigue, Marc Leone

**Affiliations:** 1 Department of Anesthesiology, Traumatology and Critical Care, Hôpital Nord, Aix-Marseille Université, Marseille, France; 2 Department of Anesthesiology and Critical Care, University Hospital, Reims, France; 3 Université Bourgogne Europe, CHU Dijon Bourgogne, Service d’anesthésie-Réanimation Et Médecine Péri-Opératoire, 21000 Dijon, France; 4 School of Medicine - La Timone Medical Campus, Aix-Marseille University, CEReSS - Health Service Research and Quality of Life Center, |27 bd Jean Moulin cedex 05, 3279 Marseille, EA France; 5 Service of Medical Biology, Hôpital Nord, Aix – Marseille University, Marseille, France; 6 Department of Anesthesiology and Surgical Care, CHU Lille, 59000 Lille, France

**Keywords:** Severe injury, Trauma induced coagulopathy

## Abstract

**Background:**

In severe trauma 30% of the patients develop a trauma-induced coagulopathy (TIC), which is associated with higher mortality, massive transfusion and prolonged intensive care stay. Conventional coagulation assays present limitations to detect TIC, primarily due to delayed turnaround time. To enhance early management of TIC viscoelastic tests have been included into guidelines as a standard of care. The Quantra device, based on sonorheometry analysis, offers a rapid bedside assessment of coagulation but lacks validation in a trauma population. The aim of this study was to validate Quantra to detect TIC compared with standard blood coagulation test in severe trauma patients.

**Methods:**

We performed a multicenter analysis of prospectively collected data (January 1, 2022–December 31, 2024) across four level-1 trauma centers. Trauma patients > 16 years underwent simultaneous Quantra and standard laboratory testing at hospital admission. Diagnostic performance for prespecified thresholds—activated partial thromboplastin time (aPTT) ratio > 1.5, prothrombin time ratio (PTr) > 1.5, fibrinogen ≤ 1.5 g/L, and platelet concentration ≤ 50, ≤ 100, and ≤ 150 G/L—was assessed by receiver operating characteristic curves in a derivation cohort, with Youden-optimized cutoffs applied to an external validation cohort to determine their specific positive and negative predictive values.

**Results:**

The derivation and validation cohorts included 285 and 219 patients, respectively. In the derivation cohort, the areas under the curve (AUCs) were 0.91 [0.84–0.98] for clot time to predict an aPTT ratio > 1.5 with a best cutoff of 144.5s; 0.82 [0.75–0.90] for clot stiffness to predict a PTr > 1.5 (best cutoff 15.4hPa); 0.87 [0.81–0.93] for fibrinogen contribution to clot stiffness to predict a fibrinogen concentration < 1.5g/L (best cutoff 1.1hPa); 0.90 [0.84–0.95] for platelet contribution to clot stiffness to predict a platelets count ≤ 100G/L (best cutoff 12.4hPa). In the validation cohort, positive predictive and negative predictive values for best cutoffs were 0.67 [0.50–0.80] and 1.00 [0.98–1.00] for clot time, 0.50 [0.39–0.61] and 0.88 [0.80–0.93] for clot stiffness, 0.58 [0.44–0.70] and 0.91 [0.85–0.95] for fibrinogen contribution to clot stiffness, 0.13 [0.24–0.99] and 0.99 [0.96–1.00] for platelets contribution to clot stiffness.

**Conclusion:**

Early Quantra analysis seems to provide rapid and reliable exclusion of TIC. Prospective investigations remain required to determine usefulness in TIC management.

**Supplementary Information:**

The online version contains supplementary material available at 10.1186/s13049-026-01610-8.

## Background

Severe trauma is a major cause of morbidity and mortality worldwide and is estimated to account for 8% of all deaths annually [1]. A large proportion of these deaths are attributable to post‑traumatic hemorrhage, which remains the leading cause of preventable death [1].

About one third of patients with severe trauma are admitted to hospital with coagulation disorders known as trauma‑induced coagulopathy (TIC). TIC is associated with mortality reaching 50%, a high likelihood of massive transfusion, and the development of complications resulting in prolonged intensive care unit (ICU) stays [2]. The management of patients with TIC remains challenging while early resuscitation aiming at stopping bleeding reduces preventable deaths [3–5].

In this context, rapid diagnosis of coagulation disorders is critical. Clinicians assess coagulation by measuring the activated partial thromboplastin time (aPTT), prothrombin time ratio (PTr), platelet count, and fibrinogen concentration [1]. These tests still represent the standard of care, but they have well-known limitations. Moreover, they capture only the initiation of clot formation and therefore provide suboptimal accuracy and clinical value [6].

Viscoelastic tests (VET)—thromboelastography (TEG) and rotational thromboelastometry (ROTEM)—have gained prominence in the care of trauma patients. These tests measure the viscoelastic properties of blood during clot formation using a pin and cup system [7, 8]. They provide a rapid, dynamic, whole‑blood appraisal of hemostasis, reduce exposure to allogeneic blood products, and enable individualized, point‑of‑care transfusion strategies [9]. Guidelines now include VET as standard in the management of severe trauma [1].

The Quantra® system (HemoSonics, Virginia, USA) is a recent device assessing clot viscoelasticity using ultrasound with Sonic Estimation of Elasticity by Resonance sonorheometry (SEER). Unlike other VET devices which use mechanical parts such as pins and cups, the Quantra device operates without direct blood contact by performing real-time ultrasound analysis of the whole blood sample, allowing assessment of clot formation time and clot stiffness. This technic minimizes mechanical artifacts and improves reliability [10]. The device has been assessed in major surgery with encouraging results and good correlations with conventional VET devices and laboratory tests [11].

To date, only few studies have evaluated the ability of Quantra to detect TIC in the early phase of severe trauma management [12–14].

The goal of this study was to determine the diagnostic performance of early Quantra parameters for identifying TIC defined by standard laboratory assays, within a large derivation cohort and an independent external validation cohort.

## Methods

### Study design and oversight

We performed a multicenter, retrospective analysis of prospectively collected data from January 1, 2022 to December 31, 2024 in four regional level‑1 trauma centers (University Hospitals of Reims, Dijon, Lille and Marseille). The study followed the STROBE (Strengthening the Reporting of Observational Studies in Epidemiology) recommendations for observational studies [15]. Of note, all severe trauma patients in the institutional organization are directly admitted to ICU.

### Eligibility criteria

Patients older than 16 years with severe trauma who, at ICU admission, underwent a Quantra analysis concomitantly with standard blood coagulation tests were eligible for inclusion. Severe trauma was defined as TRENAU A or B grades at admission [16].

### Exclusion criteria

Patients transferred from another hospital or from the emergency department and those with sampling errors (insufficient blood volume or patient identification error) were excluded from the analysis.

### Ethics

Ethical approval for this study was provided by the ethical committee of Société française d'anesthésie réanimation (SFAR) on february 22th 2022 under IRB 00010254—2022–021. Individual consent was waived; patients were informed about secondary use of their data in accordance with French law [17].

### Admission protocol

Management protocols in each center were conformed to current European guidelines for trauma care [1]. For all centers all patients were admitted directly to the ICU for initial triage. Within the first 30 min of management, they undergo comprehensive clinical, biological, radiographic, and ultrasound evaluations to guide timely transfer to whole-body CT or the operating room, as dictated by their clinical status. More details are provided in the data collection and sampling supplemental data.

### Data collection (Please see supplemental data)

#### Sampling technique and measurements (Please see supplemental data)

##### Endpoints

Abnormalities of standard blood coagulation tests were defined based on the European guidelines [1] with: (1) coagulation factor deficiency as aPTT ratio > 1.5 and / or PTr > 1.5; (2) fibrinogen deficiency as a concentration ≤ 1.5 g/L; and (3) thrombocytopenia as platelet count ≤ 50 G/L. Given the expected low incidence of platelet counts < 50 G/L, thresholds of < 100 G/L and < 150 G/L were also explored.

Values considered normal for standard laboratory tests were: aPTT ratio < 1.2, PTr < 1.2, fibrinogen > 2 g/L, and platelets > 150 G/L. Only patients meeting all these criteria were analyzed to define physiological values.

The primary objective was to evaluate the positive (PPV) and negative predictive values (NPV) of Quantra tests to detect standard coagulation test abnormalities.

First the best cut off were determined from a derivation cohort (Marseille, Hôpital Nord, historical center using the Quantra device) for each tests (1) Clot Time (CT) was use to detect for aPTT ratio > 1.5 (intrinsic pathway); Clot stiffness (CS) was use to detect a PTr > 1.5 (extrinsic pathway); Fibrinogen contribution to clot stiffness (FCS) was used to detect a fibrinogen concentration < 1.5 g/L; and Platelets contribution to clot stiffness (PCS) was used to detect a platelet count < 50, < 100, and < 150 G/L.

Next the determined cut-off for each test were applied to an external validation cohort (Reims, Dijon and Lille considered as new users centers) to determine their PPV and NPV.

Secondary objectives (in the whole cohort) were:

1. to determine Quantra values in trauma patients without any coagulation abnormalities and determined their fitness with standard normal values as defined by the device constructor. Patients without any coagulation values were defined as follow: patients presenting an aPTT ratio < 1.2, a PTr < 1.2, a fibrinogen concentration > 2 g/L and platelet concentration > 150 G/L.
2. to construct Receiving Operating Characteristics (ROC) curves for each parameter to predict coagulation abnormalities as defined above;
3. To construction ROC curves of each Quantra® parameters to predict massive transfusion defined as the need of 4 or more red blood cells in the first 6 h or 10 or more red blood cells in the first 24h after ICU admission; (supplemental data).
4. To construction ROC curves of each Quantra® parameters to predict mortality at day 30.

### Sample size

The number of patients required to construct a ROC curve with an area under the curve (AUC) of 0.75, accuracy of 0.1, and α‑risk of 5% is reported to be 50–100 depending on the event rate. We considered that 100 patients would be sufficient to construct ROC curves for an event rate ≥ 20% in line with published methods [18].

### Statistical analysis

Quantitative variables are reported as medians (25th–75th percentiles) or means ± standard deviation according to distribution; qualitative variables as counts and percentages.

A univariable analysis compared characteristics of the derivation and validation cohorts.

Using ROC curves analysis, we assessed the ability of Quantra values to detect the primary outcomes (aPTT ratio > 1.5 using CT; PTr > 1.5 using CS; fibrinogen < 1.5 g/L using FCS; platelets < 50, < 100, and < 150 G/L using PCS) in the monocentric derivation cohort. AUCs quantified accuracy. Optimal cutoffs were determined by the Youden index and then applied to the external validation cohort to estimate sensitivity, specificity, PPV, and NPV (27).

For secondary outcomes, ROC curves were constructed from the whole pooled cohort. ROC curves of ABC score have been construct as a comparative for massive transfusion evaluation.

The value distributions were tested using the Shapiro test. Physiological values in patients meeting the “normal” definition were expressed as mean ± 1.96 SD or median [2.5th–97.5th percentiles], depending on distribution. Descriptive analyses were performed with Rplusplus® (Christophe Genolini, Andeol Evain, Julien Lopez, Marc Heinrich, Released 2025–08-21, "R + + , l'essentiel" for Windows, Version 1.8.08, Toulouse, France). ROC analyses and cutoff determination used the pROC library in R version 3.2.3 (https://www.r-project.org).

## Results

### Derivation cohort

**Fig. 1:**
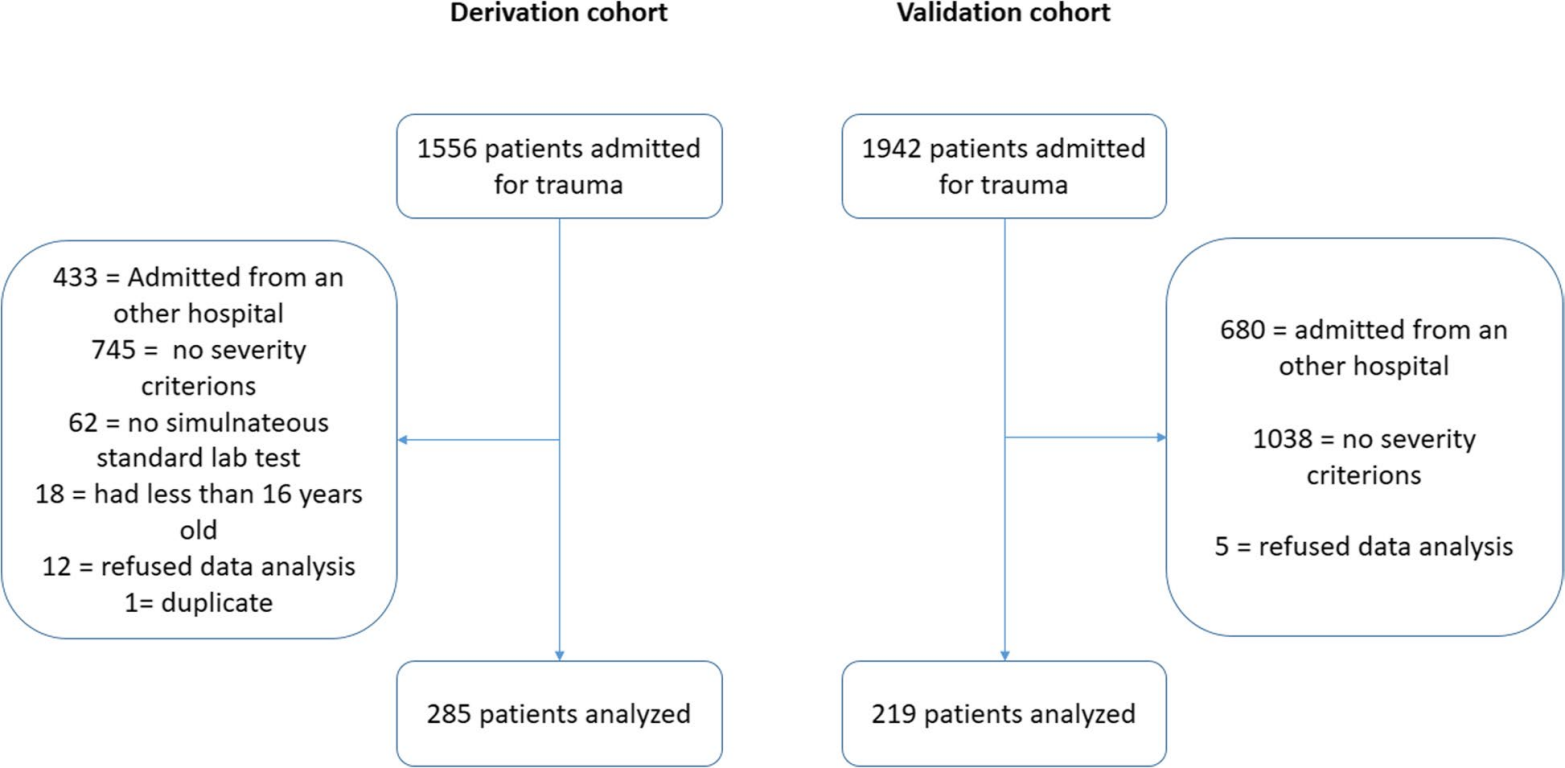
Flowchart

During the study period, 1556 patients were admitted for trauma to Nord hospital center. Of them, 427 were excluded because admitted from another hospital. Among the 1129 remaining patients, 308 met the inclusion criteria by undergoing simultaneous standard coagulation testing and Quantra analysis. After excluding five patients who refused the use of their data, and one with an identification error (duplicate), 285 patients were analyzed **(**Fig. 1**).**

### Validation cohort

During the study period, 1942 patients were admitted to the ICU for trauma. After exclusion of 1723 patients due to non-inclusion criteria, 219 patients were analyzed (Fig. 1).

### Descriptive results

The descriptive characteristics of patients and their outcomes are presented in supplemental Table 1.

The combined cohort included 386 (77%) males, with a mean age of 44 (± 20) years. Blunt trauma accounted for 419 (83%) cases. Twenty-three (5%) and 47 (10%) patients were treated with anticoagulants and antiplatelet therapy prior to admission. The mean ISS for the cohort was 26 (± 16). Massive transfusion at 6 h and 24 h occurred in 83 (16.5%) and 32 (6.4%) patients, respectively. The ICU and 30‑day mortality was reported in 117 (23%) and 124 (25%) patients, respectively (Supplemental Table 1). Univariable analysis showed no significant differences between the derivation and validation cohorts except for ASA and SAPS II scores, which were higher in the derivation cohort (Supplemental Table 1). Finally, 229 (9%) errors of tests were recorded out of 2525 tests (1 missing parameter of the panel was considered as 1 test error).

Occurrence of TIC, Quantra values and secondary endpoints are presented in the Table 1 for each cohort.

**Table 1:** Occurrence of endpoints with univariate analysis for each cohort

		**Overall *n* = 504 (100%)**	**Derivation cohort *n* = 285 (56.5%)**	**Validation cohort *n* = 219 (43.5%)**	***P* Value**
**Primary endpoints**					
aPTT Ratio		1.3 (1.1)	1.4 (1.1)	1.3 (1)	0.332
	Missing		7 (2.5)	17 (7.8)	
Occurrence of aPTT ratio > 1.5 (%)		70 (15)	42 (15)	28 (14)	0.791
PT (%)		67.9 (± 23)	69.5 (± 23)	65.7 (± 23)	0.064
	Missing		5 (1.8)	8 (3.7)	
Occurrence of PT < 50% (%)		95 (19)	44 (16)	51 (24)	51 (24)
PT Ratio		1.6 (1.5)	1.6 (1.8)	1.6 (0.9)	0.518
	Missing		7 (2.5)	9 (4.1)	
Occurrence of PT Ratio > 1.5 (%)		122 (25)	62 (22)	60 (29)	0.14
Fibrinogen (g/L)		2.1 (1.1)	2.1 (1.2)	2.1 (0.9)	0.783
	*Missing*		8 (2.8)	7 (3.2)	
Occurrence of Fibrinogen < 1.5 g/L (%)		126 (25.4)	70 (25.3)	56 (25.4)	1
Thrombocythemia (G/L)		218 (76)	223 (75)	213 (78)	0.147
	Missing		3 (1.1)	1 (0.5)	
Occurrence of Thrombocythemia < 50 G/L (%)		7 (1)	5(1.8)	2(1)	0.672
Occurrence of Thrombocythemia < 100 G/L (%)		24 (4.8)	12 (4.3)	12(5.5)	0.662
Occurrence of Thrombocythemia < 150 G/L (%)		88 (17.6)	43(15.2)	45 (20.6)	0.146
**Quantra Values**					
CT (s)		132.2 (58.3)	132.2 (56.9)	132.1 (60.1)	0.991
	Missing		25 (8.8)	4 (1.8)	
CS (hPa)		17.2 (14.9)	18.2 (19)	16.1 (7.2)	0.102
	Missing		29 (10.2)	8 (3.7)	
PCS (hPa)		15.1 (6.4)	15.3 (6.6)	15 (6.2)	0.609
	Missing		32 (11.2)	16 (7.3)	
FCS (hPa)			1.6 (1.2)	1.6 (0.9)	0.543
	Missing		30 (10.5)	15 (6.8)	
CSL (%)		96.9 (11.8)	95.9 (14.7)	98 (6.7)	0.056
	Missing		50 (17.5)	20 (9.1)	
**Secondary Endpoints**					
ABC score > 1 (%)		143 (28.8)	90 (32.5)	53 (24.2)	0.054
	Missing		8 (2.8)	0 (0)	
Massive transfusion at 6H (%)		83 (16.5)	46 (16.1)	37 (16.9)	0.916
Massive Transfusion at 24H (%)		32 (6.4)	19 (6.7)	13 (5.9)	0.865
Alive at day 30 (%)		380 (75.4)	214 (75.1)	166 (75.8)	0.937

*aPTT* Activated partial thromboplastin time, *PT* Prothrombine time, *CT* Clot time, *CS* Clot stiffness, *FCS* Fibrinogen contribution to clot stiffness, *PCS* Platelet contribution to clot stiffness

### Primary endpoint

In the derivation cohort, the best cutoff has been determined and next applied to the validation cutoff to evaluated their PPV and NPV (Table 2).

**Table 2:** AUCs value of Quantra tests on derivation cohort and performance on validation cohort

	**Derivation cohort**							**Validation cohort**			
Variables	**AUC**	**Cut-off**	**n**	**Sensibility**	**Specificity**	**PPV**	**NPV**	**Sensibility of calculated cut-off**	**Specificity of calculated cut-off**	**PPV of calculated cut-off**	**NPV of calculated cut-off**
CT (s) To predict aPTT ratio > 1.5	0.91 [0.84–0.98]	144.5	252	0.86 [0.71–0.95]	0.93 [0.89–0.96]	0.68 [0.53–0.81]	0.98 [0.94–0.99]	1.00 [0.88–1.00]	0.92 [0.87–0.95]	0.67 [0.50–0.80]	1.00 [0.98–1.00]
CS (hPa) to predict PTr > 1.5	0.82 [0.75–0.90]	15.4	249	0.74 [0.60–0.84]	0.81 [0.74–0.86]	0.53 [0.42–0.64]	0.91 [0.86–0.95]	0.76 [0.63–0.86]	0.69 [0.60–0.76]	0.50 [0.39–0.61]	0.88 [0.80–0.93]
FCS (hPa) to predict fibrinogen concentration < 1.5 g/L	0.87 [0.81–0.93]	1.1	248	0.73 [0.60–0.83]	0.89 [0.83–0.93]	0.69 [0.56–0.79]	0.91 [0.85–0.94]	0.72 [0.57–0.84]	0.84 [0.77–0.89]	0.58 [0.44–0.70]	0.91 [0.85–0.95]
PCS (hPa) to predict platelets concentration < 150 G/L	0.90 [0.84–0.95]	10.9	250	0.74 [0.57–0.88]	0.91 [0.86–0.94]	0.57 [0.41–0.71]	0.96 [0.92–0.98]	0.66 [0.49–0.80]	0.84 [0.77–0.89]	0.51 [0.37–0.65]	0.91 [0.85–0.95]
PCS (hPa) to predict platelets concentration < 100 G/L	0.94 [0.88–1.00]	12.4	250	1.00 [0.63–1.00]	0.79 [0.73–0.84]	0.13 [0.06–0.25]	1.00 [0.98–1.00]	0.90 [0.55–1.00]	0.70 [0.63–0.76]	0.13 [0.24–0.99]	0.99 [0.96–1.00]
PCS (hPa) to predict platelets concentration < 50 G/L	0.93 [0.83–1.00]	12.4	250	1.00 [0.40–1.00]	0.77 [0.71–0.82]	0.07 [0.02–0.16]	1.00 [0.98–1.00]	1.00 [0.16–1.00]	0.68 [0.61–0.74]	0.03 [0.10–1.00]	1.00 [0.97–1.00]

*PPV* Predictive positive value, *NPV* Negative predictive value, *CT* Clot time, *CS* Clot stiffness, *FCS* Fibrinogen contribution to clot stiffness, *PCS* Platelets contribution to clot stiffness

Regarding the clot time (CT), its area under the curve (AUC) was 0.91 [0.84–0.98] to predict an aPTT ratio > 1.5 with a best cutoff of 144.5s, its PPV and NPV were respectively 0.67 [0.50–0.80] and 1.00 [0.98–1.00].

The AUC for clot stiffness (CS) to predict a PTr > 1.5 was 0.82 [0.75–0.90] with a best cutoff of 15.4hPa and a PPV and NPV of 0.50 [0.39–0.61] and 0.88 [0.80–0.93] respectively.

The AUC of fibrinogen contribution to clot stiffness (FCS) to predict a fibrinogen concentration < 1.5g/L was 0.87 [0.81–0.93] with a best cutoff of 1.1hPa. The PPV and NPV were respectively 0.58 [0.44–0.70] and 0.91 [0.85–0.95].

The AUC of platelets contribution of clot stiffness (PCS) were 0.90 [0.84–0.95], 0.94 [0.88–1.00] and 0.93 [0.83–1.00] to predict a platelets concentration ≤ 150G/L, ≤ 100G/L and ≤ 50G/L respectively. Details of PPV and NPV are provided in Table 2.

### Secondary endpoints

#### Description of normal Quantra® values in trauma population

**Fig. 2:**
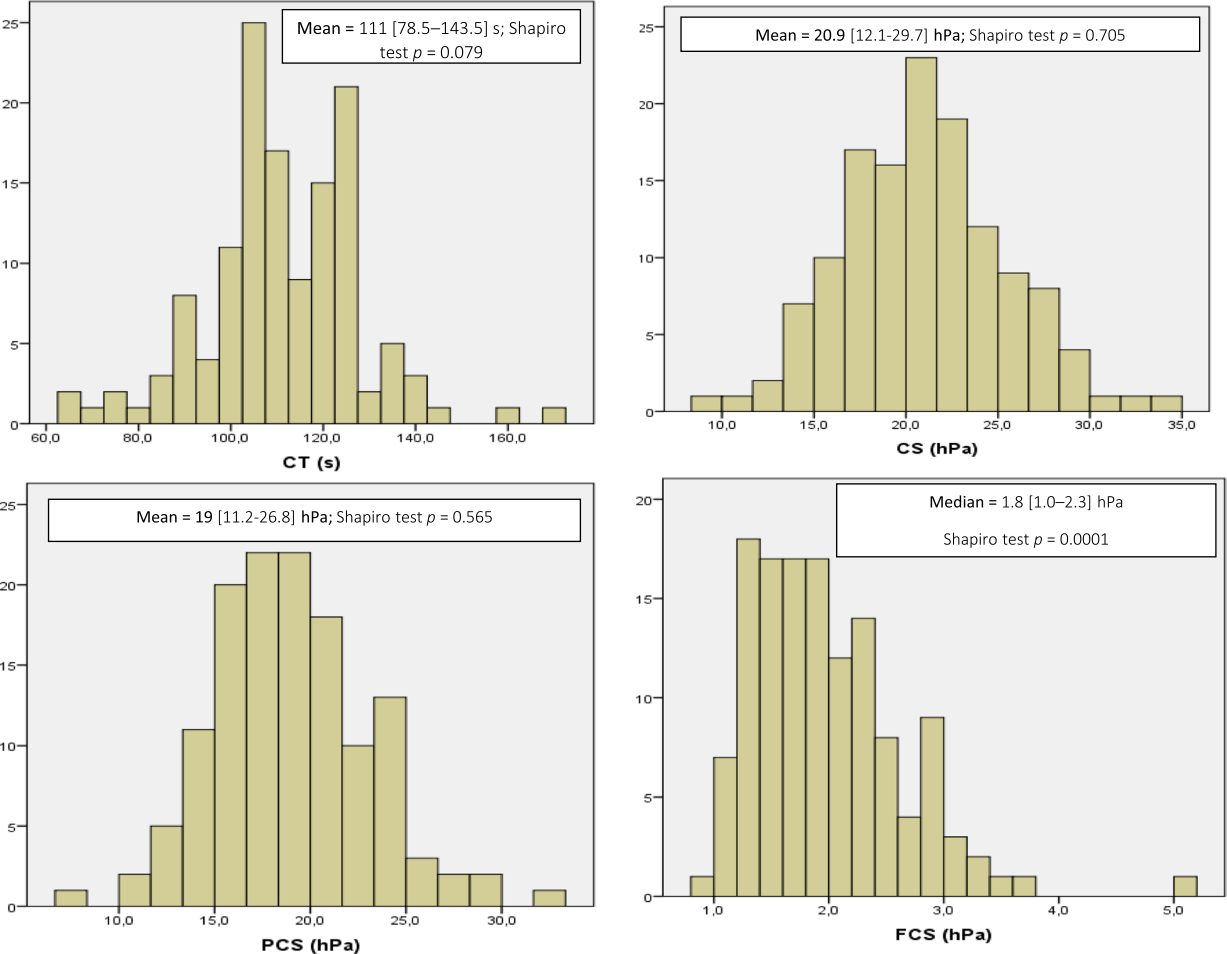
Distributions of the Quantra tests values for physiological coagulation laboratory tests. CT: clot time; CS: clot stiffness; PCS: Platelets contribution to clot stiffness; FCS: Fibrinogen contribution to clot stiffness

In the whole cohort analysis, 133 patients did not have coagulation disorders at ICU admission. Among parameters, only FCS was non‑normally distributed. Physiological values were: CT = 111 [78.5–143.5]s; CS = 20.9 [12.1–29.7]hPa; FCS = 1.8 [1.0–2.3]hPa; PCS = 19 [11.2–26.8]hPa; and (Fig. 2**).** This findings are lower that the normal values provided by device’s constructor (1) CT’s normal range: 110–166 s); (2) CS’s normal range: 13.0–33.2 hPa); (3) FCS’s normal range: 1.0–3.7 hPa); and (4) PCS: normal range, 11.9–29.8 hPa).

#### ROC curves construction for secondary endpoints

All results are summarized in the Table 3.

**Table 3:** AUCs values of Quantra tests on whole cohort to predict secondary outcomes

**Variable**s	**AUC**	**Cut-off**	**n**	**Sensibility**	**Specificity**	**PPV**	**NPV**
Traumatic induced coagulopathy thresholds							
CT (s)	0.94 [0.90–0.98]	145.5	451	0.92 [0.83–0.97]	0.93[0.90–0.95]	0.68 [0.57–0.78]	0.99 [0.97–1.00]
CS (hPa)	0.81 [0.76–0.86]	13.85	451	0.70 [0.61–0.78]	0.84 [0.80–0.88]	0.60 [0.52–0.69]	0.89 [0.85–0.92]
PCS (< 150 G/L) (hPa)	0.86 [0.82–0.90]	13.45	452	0.84 [0.74–0.92]	0.76 [0.71–0.80]	0.42 [0.34–0.50]	0.96 [0.93–0.98]
PCS (< 100 G/L) (hPa)	0.91 [0.87–0.96]	13.35	452	1.00 [0.81–1.00]	0.70 [0.66–0.75]	0.12 [0.07–0.19]	1.00 [0.99–1.00]
PCS (< 50 G/L) (hPa)	0.93 [0.85–1.00]	6.5	452	0.83 [0.36–1.00]	0.93 [0.90–0.95]	0.13 [0.04–0.28]	1.00 [0.99–1.00]
FCS (hPa)	0.87 [0.82–0.91]	0.95	452	0.69 [0.60–0.78]	0.91 [0.88–0.94]	0.72 [0.62–0.80]	0.90 [0.87–0.93]
Massive transfusion H24							
CT (s)	0.77 [0.65–0.88]	147.5	473	0.69[0.49–0.85]	0.85 [0.81–0.88]	0.23 [0.15–0.33]	0.98 [0.96–0.99]
CS (hPa)	0.80 [0.71–0.88]	11.65	465	0.73 [0.54–0.88]	0.81 [0.77–0.85]	0.21 [0.14–0.31]	0.98 [0.96–0.99]
PCS (hPa)	0.81 [0.73–0.90]	10.25	454	0.73 [0.54–0.88]	0.84 [0.80–0.88]	0.25 [0.16–0.35]	0.98 [0.96–0.99]
FCS (hPa)	0.74 [0.64–0.84]	0.75	457	0.53 [0.34–0.72]	0.87 [0.84–0.90]	0.23 [0.13–0.34]	0.96 [0.94–0.98]
ABC score	0.71 [0.61–0.80]	1.5	494	0.61 [0.42–0.78]	0.74 [0.69–0.78]	0.13[0.08–0.20]	0.97 [0.94–0.98]
Dead at day 30							
CT (s)	0.74 [0.68–0.80]	139.5	475	0.56 [0.46–0.65]	0.88 [0.84–0.91]	0.60 [0.50–0.69]	0.86 [0.82–0.89]
CS (hPa)	0.75 [0.69–0.81]	12.9	467	0.58 [0.49–0.67]	0.84 [0.80–0.88]	0.55 [0.46–0.64]	0.86 [0.82–0.89]
PCS (hPa)	0.74 [0.68–0.80]	12.45	456	0.61 [0.51–0.70]	0.81 [0.76–0.85]	0.50 [0.41–0.58]	0.87 [0.82–0.90]
FCS (hPa)	0.72 [0.66–0.78]	0.85	459	0.51 [0.42–0.61]	0.90 [0.87–0.93]	0.63 [0.52–0.73]	0.85 [0.81–0.89]
CT (s)	0.74 [0.68–0.80]	139.5	475	0.56 [0.46–0.65]	0.88 [0.84–0.91]	0.60 [0.50–0.69]	0.86 [0.82–0.89]

*PPV* Predictive positive value, *NPV* Negative predictive value, *CT* Clot time, *CS* Clot stiffness, *PCS* Platelets contribution to clot stiffness, *FCS* Fibrinogen contribution to clot stiffness

## Discussion

We evaluated the ability of Quantra device to detect standard blood coagulation test disorders in severe trauma patients. The ROC analyses suggested that the device provides excellent NPV to rule out coagulation disorders.

To our knowledge, with a total of 504 patients analyzed this is the largest cohort using this device for early detection of TIC. Only one retrospective study including 156 patients previously explored its ability to detect TIC [12]. In a multicenter study, Moore et al*.* compared Quantra with conventional VET platforms in 259 trauma patients and reported moderate‑to‑strong correlations (*r* = 0.64 – 0.88), concluding that Quantra conveys equivalent information to ROTEM delta and TEG 6s for trauma patients [14]. In another observational study including 209 patients, Rossetto et al*.* found strong correlations between Quantra and ROTEM parameters regarding CS with EXTEM (specific extrinsic pathway coagulation test) clot amplitude at 5 min (*r* = 0.90), fibrinogen contribution with FIBTEM (specific fibrinogen concentration) test (*r* = 0.85), and platelet contribution with EXTEM-FIBTEM test (*r* = 0.73) [13].

Focusing on the ability to detect TIC, our findings from Quantra device were superposable to other VET devices. Rosetto et al. found that this device was better than ROTEM to detect TIC defined by an INR > 1.2 (AUC = 0.83 vs 0.79, *p* = 0.038) [13]. In a meta-analysis, the ROTEM device has shown a range between 0.7 and 1 of sensitivity and 0.58 to 1 of specificity to detect a TIC depending of the EXTEM clot amplitude values at five, 10 or 15 min of testing [19]. Using TEG, Nascimento et al. found a low sensitivity (0.33) and a high specificity (0.95) to detect coagulopathy defined by an INR of 1.5 or more [20].

With an AUC of 0.87, this cohort highlights a good ability of the Quantra device to detect low concentration of fibrinogen in severe trauma patients. Previous studies reported the ability of conventional VET devices to detect fibrinogen deficit in trauma [1, 21]. A review reported AUCs ranging from 0.70 to 0.95 depending on the fibrinogen threshold (1.0, 1.5, or 2.0 g/L) [21]. Similar results have been shown in a study with an ability of FCS to detect fibrinogen ≤ 2 g/L with an AUCs of 0.79, a NPV of 0.88 and a specificity of 0.60 [13].

With respect to thrombocytopenia, with AUCs ranging from 0.90 to 0.94 depending of the cutoff of platelet counts, the results of this cohort seem to be in line with conventional VET devices and available data. Only a few studies have assessed VET thresholds for thrombocytopenia in trauma. A large observational study reported a sensitivity at 0.7 to detect a platelet count ≤ 100 G/L [22]. Rugeri et al*.* showed that ROTEM detected platelets ≤ 50 G/L with a sensitivity at 1 and a specificity at 0.83 [23]. In a 209 patients, Roseto et al*.* did not find differences between ROTEM and Quantra to detect platelet count < 150G/L [13]. In the Roseto et al. study, Quantra had an AUC of 0.87 [0.81 – 0.93] [13].

Focusing on normal values definition for trauma patients, the results are notably lower than those given by the manufacturer. This may be due to the procoagulant profiles of patients with no severe injury [24, 25]. Moreover, these values were outside the range of those patients with a TIC except for the CS and PCS tests. In a previous cohort of trauma patients, Quantra showed cut-off for TIC detection in the ranges of the constructor normal values [12].

Regarding the massive transfusion, the predictive values of the Quantra device were similar from those of the ABC score. This result suggest that Quantra may be useful to predict massive transfusion needs. However, in a large RCT, no evidence was found that transfusion guided on VET was associated with a reduction of massive transfusion [26].

Regarding the prediction of mortality at day 30, the analysis based on Quantra results predicted the occurrence of death with an AUC of 0.72–0.75. This result is insufficient to establish a reliable predictive criterion, particularly because the retrospective design of the study does not clarify how Quantra analysis results influenced patient management. Nevertheless, existing data demonstrate an association between viscoelastic test results and patient severity. The occurrence of death at 6 h is predicted with an AUC of CT of 0.92, which was similar to that of the EXTEM A5 test from the ROTEM [13]. In a large multicentric cohort of 1499 patients, Murali et al*.* found a correlation of a constructed TIC score and mortality from VET values (1103 from TEG and 396 from ROTEM). They found an increase of 53% of mortality risk for an increase of 1 point of the constructed TIC score with an odds ratio at 1.53, and a 95% confidence interval from 1.33 to 1.76 (*P* < 0.001) [27].

This study has several limitations. Its retrospective design may have introduced biases we could not fully account for. We mitigated this by using prospectively collected data and analyzing samples drawn before any hemostatic therapy (except tranexamic acid). Second, CS was used as a surrogate for PTr to explore extrinsic pathway, which cannot be interpreted without considering fibrinogen and platelet counts. Third, the Quantra use was not mandatory during the study period, focusing on the more severe patients resulting in a substantial proportion of admissions without testing; this may limit generalizability, particularly among stable patients. Fourthly, we reported data for severe TIC thresholds and normal coagulation test results but did not include patients with mild to moderate coagulopathy (e.g., aPTT ratio and PTr between 1.2–1.5, or fibrinogen concentration between 1.5 and 2.0 g/L). This choice leads to a lack of data for patients with middle traumatic coagulopathy. Next, although the practices were based on European guidelines, we cannot be certain that the transfusion thresholds were similar in all centers. Sociocultural determinants of health (including ethnicity) were unavailable per local regulations, which may limit assessment of generalizability.

## Conclusion

This findings suggest that Quantra is a relevant tool for early detection of coagulation disorders in trauma. It showed satisfactory performance in identifying and excluding abnormalities in standard coagulation tests, although diagnostic thresholds still lie within or near the normal ranges.

## Supplementary Information

Additional file 1.

Additional file 2.

## Data Availability

The datasets used and/or analysed during the current study are available from the corresponding author on reasonable request.

## Abbreviations

ABC: Assessment of Blood Consumption score
AIS: Abbreviated Injury Scale
ASA: American Society of Anesthesiologists
AUC: Area under the curve
CA: Cardiac arrest
CS: Clot stiffness
CSL: Clot stability to lysis
CT: Clot time
FAST: Focused assessment with sonography for trauma
FCS: Fibrinogen contribution to clot stiffness
hPa: Hectopascal
INR: International normalized ratio
ISS: Injury Severity Score
SBP: Systolic blood pressure
PCS: Platelet contribution to clot stiffness
FFP: Fresh frozen plasma
ROC: Receiver operating characteristic
ROTEM: Rotational thromboelastometry
ARDS: Acute respiratory distress syndrome
SEER: Sonic estimation of elasticity via resonance
SOFA: Sequential Organ Failure Assessment
STROBE: Strengthening the Reporting of Observational Studies in Epidemiology
aPTT: Activated partial thromboplastin time
TEG: Thromboelastography
MT: Massive transfusion
PTr: Prothrombin time ratio
ICU: Intensive care unit
VET: Viscoelastic tests
NPV: Negative predictive value
PPV: Positive predictive value
