# Supplementary material for: Sonorheometry to detect traumatic induced coagulopathy during initial management of trauma patients: an observational, multi-center study
Source: Scand J Trauma Resusc Emerg Med. 2026 Apr 15;34:91. doi: 10.1186/s13049-026-01610-8 (PMC13191944; doi:10.1186/s13049-026-01610-8)
Supplement: Supplementary file 1 — Additional file 1. [file 13049_2026_1610_MOESM1_ESM.docx]

*Supplemental data*

*Sampling technique and measurements (supplemental data)*

At admission, whole‑blood samples were systematically collected from an arterial line into 2.7‑mL citrated tubes (BD Vacutainer) according to international recommendations and local protocol. When patient was considered as severe, he underwent coagulation assessment using a VET device (Quantra system) in addition of standard laboratory testing ^1^. Severity criterions to perform a SEER analysis was based on TRENEAU classification^2^. All patients with TRENAU A grade received a SEER analysis. Patients classified with TRENAU B grade received a SEER analysis at the physician discretion. Prediction of massive transfusion protocol were based on ABC and Redflag scores^3,4^.

Four Quantra parameters were analyzed: (1) initiation of clot time in seconds (CT; manufacturer’s normal range, 110–166 s); (2) clot stiffness in hectopascal (CS; normal range, 13.0–33.2 hPa); (3) fibrinogen contribution to clot stiffness (FCS; normal range, 1.0–3.7 hPa); and (4) platelet contribution to CS (PCS; normal range, 11.9–29.8 hPa). Multivial cartridges (QStat) were used for analysis. In whole blood, CT is measured using kaolin, an activator of the intrinsic pathway ^5^. It reflects the functional status of coagulation factors leading to fibrin formation and relates to factor concentrations in the intrinsic pathway. CS is measured in the presence of thromboplastin (an activator of the extrinsic pathway) and polybrene (a heparin inhibitor) and integrates information on extrinsic coagulation factors, fibrinogen concentration, and platelet count ^5,6^. FCS is measured in the presence of thromboplastin and polybrene with the addition of a high dose of the antiplatelet agent abciximab to inhibit platelet function. PCS is calculated as CS minus FCS ^5^. The device can also analyze clot stability to lysis (CSL; percentage; normal range, 93%–100%) and detect potential hyperfibrinolysis using a dedicated QStat channel. CSL is expressed as the normalized difference between CS over time in the absence versus presence of tranexamic acid during analysis ^5^.

Standard laboratory coagulation tests included prothrombin time/international normalized ratio (INR), aPTT ratio versus control, Clauss fibrinogen assay, and platelet count performed as rapidly as possible after arterial sampling.

*Data collection (supplemental data)*

Data extracted from medical records included demographics (age, sex, weight, height) and use of anticoagulants and/or antithrombotic agents prior to admission (drug class specified).

Clinical variables comprised prehospital and admission hemodynamics (systolic and diastolic blood pressure, heart rate), occurrence of cardiac arrest, Glasgow Coma Scale score, use of invasive mechanical ventilation, and initial body temperature. Severity scores computed at admission included SOFA (Sequential Organ Failure Assessment), ISS (Injury Severity Score), ASA (American society of anesthesiologist), SAPS II (Simplified acute physiology score) and the Assessment of Blood Consumption (ABC) score ^4^ .

The ABC score was calculated for each patient using the worst prehospital and admission values ^4^. An ABC score >1 (scale 0–4) is associated with a high probability of massive transfusion. Massive transfusion was defined as transfusion of >4 units of red blood cells in the six hours after ICU admission or a transfusion of ≥10 units in the 24 hours since admission. Trauma characteristics were recorded, including mechanism (road traffic collision, fall, penetrating trauma, other) and the presence of free fluid on initial Focused Assessment Sonography for Trauma (FAST) ^4,7^.

Laboratory evaluation included complete blood count, coagulation profile (as above), serum creatinine, bilirubin, and arterial lactate on the first sample collected at ICU admission. Transfusion management was summarized by the number of blood products administered (red blood cells, plasma, platelets, fibrinogen concentrate) and the total volume of intravenous fluids in the first 24 hours. Other therapies (norepinephrine, tranexamic acid, invasive mechanical ventilation) were also recorded.

Outcome data included ICU and hospital length of stay and thromboembolic events. Vital status was assessed at ICU discharge and 30 days after admission. In addition, the number of Quantra® test failure was recorded and reported relative to the total number of tests performed per patient (each Quantra analysis gives five tests/parameters per patients: CT, CS, FCS, PCS, CSL).

References:

1. Rossaint R, Afshari A, Bouillon B, et al. The European guideline on management of major bleeding and coagulopathy following trauma: sixth edition. *Critical Care* 2023;27(1):80.

2. Bouzat P, Ageron F-X, Brun J, et al. A regional trauma system to optimize the pre-hospital triage of trauma patients. *Crit Care* 2015;19(1):111.

3. Hamada SR, Rosa A, Gauss T, et al. Development and validation of a pre-hospital “Red Flag” alert for activation of intra-hospital haemorrhage control response in blunt trauma. *Crit Care* 2018;22(1):113.

4. Nunez TC, Voskresensky IV, Dossett LA, Shinall R, Dutton WD, Cotton BA. Early prediction of massive transfusion in trauma: simple as ABC (assessment of blood consumption)? *J Trauma* 2009;66(2):346–352.

5. Ferrante EA, Blasier KR, Givens TB, Lloyd CA, Fischer TJ, Viola F. A Novel Device for the Evaluation of Hemostatic Function in Critical Care Settings. *Anesth Analg* 2016;123(6):1372–1379.

6. Hochleitner G, Sutor K, Levett C, Leyser H, Schlimp CJ, Solomon C. Revisiting Hartert’s 1962 Calculation of the Physical Constants of Thrombelastography. *Clin Appl Thromb Hemost* 2017;23(3):201–210.

7. Hamada SR, Delhaye N, Kerever S, Harrois A, Duranteau J. Integrating eFAST in the initial management of stable trauma patients: the end of plain film radiography. *Ann Intensive Care* 2016;6(1):62.
