# Supplementary material for: Sonorheometry to detect traumatic induced coagulopathy during initial management of trauma patients: an observational, multi-center study
Source: Scand J Trauma Resusc Emerg Med. 2026 Apr 15;34:91. doi: 10.1186/s13049-026-01610-8 (PMC13191944; doi:10.1186/s13049-026-01610-8)
Supplement: Supplementary file 2 — Additional file 2. [file 13049_2026_1610_MOESM2_ESM.docx]

| Supplemental Table 1 : characteristics of the cohorts | | | | | |
| --- | --- | --- | --- | --- | --- |
|  |  | **Overall n=504 (100%)** | **Derivation cohort n=285 (56.5%)** | **Validation cohort n=219 (43.5%)** | **P Value** |
| Age (years) | | 43.8 (20.0) | 43.9 (20.1) | 43.8 (19.9) | 0.94 |
| Male gender (%) | | 386 (76.6) | 220 (77.2) | 166 (75.8) | 0.795 |
| Body mass index (kg/m²) | | 25.2 (4.2) | 25.4 (4.3) | 24.9 (4.1) | 0.233 |
|  | Missing |  | 28 (9.8) | 8 (3.7) |  |
| Penetrating trauma (%) | | 86 (17.1) | 46 (16.1) | 40 (18.3) | 0.314 |
| ASA (%) | |  |  |  | 0.023 |
|  | 1 | 261 (51.9) | 148 (51.9) | 113 (51.8) |  |
|  | 2 | 170 (33.8) | 105 (36.8) | 65 (29.8) |  |
|  | 3 | 60 (11.9) | 24 (8.4) | 36 (16.5) |  |
|  | 4 | 7 (1.4) | 3 (1.1) | 4 (1.8) |  |
|  | 5 | 4 (0.8) | 4 (1.4) | 0 (0) |  |
|  | Missing |  | 1 (0.3) | 1 (0.5) |  |
| Anticoagulant medication (%) | | 23 (4.6) | 15 (5.3) | 8 (3.7) | 0.498 |
|  | Missing |  | 4 (1.4) | 0 (0) |  |
| Antithrombotic medication (%) | | 47 (9.7) | 25 (8.9) | 22 (10.1) | 0.777 |
| Missing | |  | 4 (1.4) | (0) |  |
| **Prehospital setting** | | | | | |
| First Glasgow scale score | | 10.7 (4.9) | 10.4 (4.9) | 11 (4.8) | 0.173 |
| Cardiac arrest | | 51 (10.1) | 35 (12.3) | 16 (7.3) | 0.091 |
| Minimal Systolic blood pressure (mmHg) | | 103.3 (48.2) | 99.5 (49.8) | 109 (45.4) | 0.036 |
|  | Missing |  | 19 (6.7) | 36 (16.4) |  |
| First hemoglobin (g/dL) | | 12.8 (2.3) | 12.9 (2.3) | 12.7 (2.4) | 0.334 |
|  | Missing |  | 30 (10.5) | 51 (23.3) |  |
| Fluid expansion (mL) | | 888.6 (694.6) | 855.8 (654.8) | 931.5 (742.9) | 0.255 |
|  | Missing |  | 25 (8.8) | 20 (9.1) |  |
| Vasopressor use | | 185 (36.9) | 115 (40.5) | 70 (32.3) | 0.072 |
|  | Missing |  | 1 (0.4) | 2 (0.9) |  |
| **Hospital Admission** | | | | | |
| Heart rate (bpm) | | 97 (28.1) | 95.3 (29.5) | 99.1 (26.2) | 0.128 |
| Systolic blood pressure (mmHg) | | 109.5 (34.4) | 109 (35.5) | 110.2 (33) | 0.693 |
| Norepinephrine use (%) | | 228 (45.5) | 123 (43.6) | 105 (47.9) | 0.382 |
|  | Missing |  | 3 (1.1) | 0 (0) |  |
| Mechanical ventilation (%) | | 291 (57.9) | 160 (56.3) | 131 (59.8) | 0.489 |
| Lactatemia (mmol/L) | | 4 (3.9) | 4.2 (4.1) | 3.8 (3.7) | 0.251 |
|  | Missing |  | 2 (0.7) | 17 (7.8) |  |
| Hemoglobin (g/dL) | | 12.3 (17.4) | 11.6 (2.2) | 13.1 (26.2) | 0.403 |
| Ionized Calcemia (mmol/L) | | 1.1 (0.1) | 1.1 (0.1) | 1.1 (0.1) | 0.966 |
|  | Missing |  | 18 (6.3) | 20 (9.1) |  |
| Creatinemia (µmol/L) | | 92.9 (44.1) | 92.2 (46.1) | 94 (40.8) | 0.648 |
|  | Missing |  | 4 (1.4) | 35 (16.0) |  |
| Bilirubinemia (mg/L) | | 8.5 (8.2) | 7.2 (4.7) | 10.3 (11.1) | < 0.001 |
|  | Missing |  | 5 (1.8) | 8 (3.7) |  |
| Alcoolemia (g/L) | | 0.3 (0.7) | 0.3 (0.8) | 0.3 (0.6) | 0.4 |
|  | Missing |  | 16 (5.6) | 26 (11.9) |  |
| Tranexamic use at hospital admission | | 443 (89.0) | 260 (93.2) | 183 (83.6) | 0.001 |
|  | Missing |  | 6 (2.1) | 0 (0) |  |
| SAPS II | | 45.9 (23.8) | 48.3 (24.5) | 42.8 (22.6) | 0.009 |
| SOFA score | | 7.1 (4.7) | 7.1 (4.7) | 7.1 (4.6) | 0.988 |
|  | Missing |  | 2 (0.7) | 0 (0) |  |
| ISS | | 26.4 (16.5) | 27.2 (18.1) | 25.3 (14.1) | 0.182 |
| Fluid volume infusion (mL) H24 | | 3470 (2821) | 3717 (3275) | 3282 (2409) | 0.159 |
|  | Missing |  | 12 (4.2) | 9 (4.1) |  |
| RBC at 6H (unit) | | 2.2 (3.5) | 2.3 (3.9) | 2 (3) | 0.336 |
| FFP at 6H (unit) | | 2 (3.2) | 2.7 (4) | 1.5 (2.3) | 0.001 |
|  |  |  |  |  |  |
| Platelets concentrate at 6H (unit) | | 0.4 (0.9) | 0.4 (0.9) | 0.3 (1) | 0.296 |
|  |  |  |  |  |  |
| RBC at 24H (unit) | | 2.7 (4.3) | 2.5 (4.2) | 2.9 (4.4) | 0.234 |
|  |  |  |  |  |  |
| FFP at 24H (unit) | | 2.4 (4) | 2.8 (4.3) | 2.2 (3.7) | 0.160 |
| Platelets concentrate at 24H (unit) | | 0.4 (1) | 0.4 (0.9) | 0.4 (1.1) | 0.999 |
| Thromboembolic event | | 28 (5.6) | 18 (6.3) | 10 (4.6) | 0.521 |
|  | Missing |  | 0 (0) | 1 (0.5) |  |
| Length of stay in ICU (days) | | 10.9 (15.8) | 10.3 (14.8) | 11.6 (17) | 0.388 |
| Length of stay in Hospital (days) | | 20.2 (22.1) | 18.6 (20.6) | 22.2 (23.9) | 0.076 |
| Death in ICU (%) | | 117 (23.2) | 66 (23.2) | 51 (23.3) | 1 |
| Alive at day 30 (%) | | 380 (75.4) | 214 (75.1) | 166 (75.8) | 0.937 |

*ASA : American society of anesthesiologist ; PT : Prothrombine time; aPTT: Activated partial thromboplastin time; CT : Clot time; CS : Clot stiffness; FCS: Fibrinogen contribution to clot stiffness; PCS: Platelet contribution to clot stiffness; CSL: Clot stability to lysis; SAPS : Simplified acute physiology score; SOFA : Sequential organ failure assessment; ISS : Injury severity score; RBC : Red blood cells; FFP : Fresh frozen plasma; ICU : intensive care unit.*
